# Dental Pulp Defence and Repair Mechanisms in Dental Caries

**DOI:** 10.1155/2015/230251

**Published:** 2015-10-11

**Authors:** Jean-Christophe Farges, Brigitte Alliot-Licht, Emmanuelle Renard, Maxime Ducret, Alexis Gaudin, Anthony J. Smith, Paul R. Cooper

**Affiliations:** ^1^Institut de Biologie et Chimie des Protéines, Laboratoire de Biologie Tissulaire et Ingénierie Thérapeutique, UMR 5305 CNRS, Université Lyon 1, 69367 Lyon, France; ^2^Faculté d'Odontologie, Université de Lyon, Université Lyon 1, 69372 Lyon, France; ^3^Hospices Civils de Lyon, Service de Consultations et de Traitements Dentaires, 69008 Lyon, France; ^4^INSERM UMR 1064, Centre de Recherche en Transplantation et Immunologie, Université de Nantes, Faculté d'Odontologie, 44042 Nantes, France; ^5^Oral Biology, School of Dentistry, College of Medical and Dental Sciences, University of Birmingham, Birmingham B4 6NN, UK

## Abstract

Dental caries is a chronic infectious disease resulting from the penetration of oral bacteria into the enamel and dentin. Microorganisms subsequently trigger inflammatory responses in the dental pulp. These events can lead to pulp healing if the infection is not too severe following the removal of diseased enamel and dentin tissues and clinical restoration of the tooth. However, chronic inflammation often persists in the pulp despite treatment, inducing permanent loss of normal tissue and reducing innate repair capacities. For complete tooth healing the formation of a reactionary/reparative dentin barrier to distance and protect the pulp from infectious agents and restorative materials is required. Clinical and *in vitro* experimental data clearly indicate that dentin barrier formation only occurs when pulp inflammation and infection are minimised, thus enabling reestablishment of tissue homeostasis and health. Therefore, promoting the resolution of pulp inflammation may provide a valuable therapeutic opportunity to ensure the sustainability of dental treatments. This paper focusses on key cellular and molecular mechanisms involved in pulp responses to bacteria and in the pulpal transition between caries-induced inflammation and dentinogenic-based repair. We report, using selected examples, different strategies potentially used by odontoblasts and specialized immune cells to combat dentin-invading bacteria *in vivo*.

## 1. Odontoblasts in the Dental Pulp's Defence against Caries

The crowns of erupted human teeth are covered by symbiotic microbial communities, mainly composed of Gram-positive saprophytic bacteria which are normally harmless to the tooth. These communities adhere as biofilms to the highly mineralized enamel that constitutes a barrier which is impermeable to microorganisms and protects the underlying mineralized dentin and the loose connective tissue situated at the centre of the tooth, the dental pulp. However, when placed in a sugar-rich environment, specific bacterial populations from these communities release acids that progressively demineralize enamel [1, 2]. This leads to the appearance of a carious lesion characterized by a cavity within which “cariogenic” bacteria proliferate and release additional acids that progressively deepen the lesion. When the enamel barrier is disrupted, dentin becomes degraded by Gram-positive bacteria, including streptococci, lactobacilli, and actinomyces that largely dominate the dentin caries microflora [3]. The proliferation and metabolic activity of these microorganisms lead to the release of bacterial components into dentinal tubules and their diffusion towards the peripheral pulp. Dentin demineralization may also enable the release of bioactive molecules from the dentin matrix [4]. Recognition of bacterial components by host cells at the dentin-pulp interface triggers host protective events including antibacterial, immune, and inflammatory responses. These events may eliminate early stage bacterial infection and block the route of its progression when accompanied by dentin formation at the pulp-dentin interface. Unchecked, bacterial invasion results in irreversible chronic pulp inflammation, most often after a long phase of chronic inflammation. Subsequently, pulp necrosis, infection of the root canal system, and periapical disease may occur [3, 5]. Pulp inflammation, also called “pulpitis,” generally dampens after microorganism removal by the dental practitioner and neutralization of intratubular diffusing components by the pulp immune system, both decreasing the production of proinflammatory mediators [6]. However, when the caries lesion is close to the dentin-pulp interface, pulpal inflammation does not resolve completely after dental treatment and may become low-grade and chronic in nature. This chronic inflammation is responsible, as in other connective tissues, for the permanent loss of normal tissue function and the reduction of defence capacities to future injuries. On occasions, rapid cessation of inflammation enables complete pulp healing with the formation of a barrier of reactionary dentin by the original surviving odontoblasts and/or reparative dentin by newly differentiated odontoblast-like cells in animal models [7]. Dentin neoformation protects the underlying pulp from the dentin infection and the crown filling biomaterial, thus reducing the risk of permanent irritation by external bacterial or chemical agents. It is reasonable to speculate that rapid reactionary/reparative dentin formation is initiated, the quicker pulp healing occurs, and health is reestablished. So, from a clinical point of view, it appears crucial to identify molecular and cellular agents able to dampen immune/inflammatory events within the dental pulp and promote rapid return to tissue homeostasis and health once the bacterial infection is resolved [2, 8–10]. Such agents should help to prevent the evolution of the pulp inflammation towards becoming chronic in nature. To identify these agents, it is important to gain an in-depth knowledge of the events that initiate and control the early steps of human pulp antibacterial defence and dentinogenesis-based reparative mechanisms in caries-affected human teeth. This paper focusses on key cellular and molecular mechanisms involved in pulp responses to bacteria and in the pulpal transition between caries-induced inflammation and dentinogenic-based repair. We report, using selected examples, different strategies potentially used by odontoblasts and specialized immune cells to combat dentin-invading bacteria* in vivo*.

Odontoblasts are the first pulpal cells encountered by dentin-invading pathogens and their released products owing to both their specific localization at the pulp-dentin interface and the embedding of their long cellular processes in dentin tubules. We and others have therefore hypothesized that, in the tooth, they represent the first biologically active line of defence for the host, fulfilling the role devoted elsewhere in the body to skin and mucosal epithelial cells [12, 13]. Odontoblasts may thus be involved in combatting bacterial invasion and activating innate and adaptive aspects of dental pulp immunity. Both these events can only be activated following pathogen recognition by pulp cells. In a general way, such recognition occurs through the detection (“sensing”) of molecular structures shared by pathogens and that are essential for microorganism survival. These structures are termed Pathogen-Associated Molecular Patterns (PAMPs) and are sensed by a limited number of so-called Pattern-Recognition Receptors (PRRs). One important class of PRRs is represented by the Toll-like receptor (TLR) family that is crucial for the triggering of the effector phase of the innate immune response [14–16]. TLR2 and TLR4, which are involved in Gram-positive and Gram-negative bacterial sensing, respectively, have been previously detected in the odontoblast cell membrane in healthy pulp, indicating that odontoblasts are equipped to recognize these pathogens when they diffuse through dentin tubules during the carious infection [13, 17]. TLR2 has been shown to be upregulated in odontoblasts beneath caries lesions compared with odontoblasts beneath healthy dentin [2], suggesting that these cells are not only adapted to the recognition of Gram-positive bacteria but that they are also able to amplify their response to these pathogens.

One major consequence of TLR activation is upregulation of innate immunity effectors, including antimicrobial agents and proinflammatory cytokines and chemokines that recruit and activate tissue resident and blood borne immune/inflammatory cells [18, 19]. Odontoblasts have been found to produce several antibacterial agents, among which beta-defensins and nitric oxide have received particular attention. Beta-defensins (BDs) are cationic, broad-spectrum antimicrobial peptides that kill microorganisms by forming channel-like micropores that disrupt membrane integrity and induce leakage of the cell content [20–23]. They are mainly produced by epithelial and immune cells to protect skin and internal mucosae from pathogen invasion. Whereas BD-1 is generally constitutively expressed, BD-2, BD-3, and BD-4 are induced by microorganisms that come into contact with host cells. Several* in vitro* studies have reported that BDs might also be involved in the pulpal defence against caries-related microorganisms. Indeed, BD-2 was shown to possess antibacterial activity against* S. mutans* and* L. casei* [24–26] and BD-3 exhibited antibacterial activity against mature biofilms containing* Actinomyces naeslundii*,* Lactobacillus salivarius*,* Streptococcus mutans*, and* Enterococcus faecalis* [27]. A proinflammatory role was also proposed for BD-2, which upregulates interleukin (IL-) 6 and as Chemokine [C-X-C Motif] Ligand 8 (CXCL8, also known as IL-8) in odontoblast-like cells* in vitro* [28]. A positive feedback mechanism could exist between inflammatory cytokines and BD-2, the expression of which was found to be stimulated by IL-1*α* and tumor necrosis factor (TNF-) *α* in cultured human dental pulp cells [29, 30]. The proinflammatory effect of BD-2 could be augmented by the fact that it chemoattracts immature antigen-presenting dendritic cells (DCs), macrophages, CD4+ memory T cells, and natural killer (NK) cells by binding to cell surface chemokine receptors [22].* In vitro*, odontoblast BD-2 gene expression was not modified by TLR2 activation in a tooth organ culture model, whereas BD-1 and BD-3 genes were downregulated [13]. BD-2 gene expression was upregulated upon TLR4 activation, which suggests that BDs are differentially produced by odontoblasts to combat Gram-positive and Gram-negative bacteria.* In vivo* studies have revealed that odontoblasts in healthy pulp synthesize BD-1 and, to a lesser extent, BD-2 [31, 32]. Constitutive expression of low levels of BDs in the odontoblast layer might be necessary to destroy individual or very small groups of oral early stage bacterial invaders which enter the tooth through tiny, clinically undetectable lesions such as enamel cracks, before these bacteria engage with the pulpal immune system. Discrepancies exist between reports regarding the regulation of BDs in inflamed dental pulp. Indeed, BD-1 and BD-2 were first reported to be decreased during irreversible pulpitis [28], whereas, in a more recent study, BD-1 and BD-4 were found to be increased in inflamed pulps compared with healthy ones; the expression of BD-2 and BD-3 however remained constant [32]. Differences in the inflammatory status between pulp samples (reversible versus irreversible inflammation) may be responsible for these discrepancies. It remains unclear as to whether BDs are present in the bacteria-challenged inflamed pulp at levels that enable them to play a major role in the tissue defence against dentin-invading bacteria. Further studies are needed to investigate the antibacterial activity of BDs produced at* in vivo* relevant concentrations by odontoblasts challenged with caries-related microorganisms. Another important antimicrobial agent produced by odontoblasts challenged with microbial components is nitric oxide (NO). NO is a potent antibacterial, highly diffusible free radical produced from L-arginine through oxidation by NO synthases (NOS), of which there are 3 isoforms: NOS1 (neuronal NOS) and NOS3 (endothelial NOS), that are constitutively expressed in most healthy tissues, and NOS2 (inducible NOS), generally absent from healthy tissues and induced in particular in tissues challenged by microorganisms. NOS1 and NOS3 are constitutively expressed in physiological conditions by many cells and produce very low, picomolar to nanomolar range NO concentrations within seconds or minutes. NOS2 is mostly involved in host defence by producing high, micromolar range amounts of NO for sustained periods of time (hours to days) [33–39]. NOS2 is not, or only moderately, expressed in healthy human dental pulps and was found to be rapidly upregulated in inflamed pulps [40–44]. Furthermore, NOS2 activation was shown to promote the accumulation of neutrophils and macrophages in experimentally inflamed rat incisor pulps [42, 43]. CXCL8 might also be involved in this process since NO has been shown to stimulate the production of this chemokine in human pulp cells* in vitro* [45]. Human odontoblasts in the inflamed dental pulp showed a marked immunoreactivity for 3-nitrotyrosine (a biomarker for NO-derived peroxynitrite), suggesting that these cells release NO upon NOS2 activation [44]. Indeed, NO release might constitute an important defence mechanism against* Streptococcus mutans* as the growth of these microorganisms has been shown to be inhibited by NO* in vitro* [46]. Accordingly, NO produced at high concentration by NOS2 in the inflamed pulp might be used by odontoblasts as a weapon to combat cariogenic bacteria. We have recently presented evidence that odontoblasts differentiated* in vitro* strongly amplify their NOS2 synthesis and NO production upon TLR2 activation. The NO produced was found to inhibit the growth of* Streptococcus mutans*, thus suggesting the role of this odontoblast-derived molecule in the limitation of the intradentinal progression of caries-related microorganisms [47].

Numerous* in vitro* studies have also shown that odontoblasts produce inflammatory cytokines and chemokines when challenged by PAMPs from Gram-positive bacteria [12, 13]. In particular, odontoblasts differentiated* in vitro* were found to be responsive to lipoteichoic acid (LTA), a Gram-positive bacteria wall component recognized at the cell surface through TLR2. Engagement of odontoblast TLR2 by LTA upregulated TLR2 itself and NOD2, a cytosolic PRR, which led to nuclear factor-*κ*B (NF-*κ*B) and p38 mitogen-activated protein kinase (MAPK) signalling activation, dentinogenesis inhibition, and production of the proinflammatory chemokines Chemokine [C-C Motif] Ligand 2 (CCL2), CXCL1, CXCL2, CXCL8, and CXCL10 [2, 12, 48–51]. Chemokine production by odontoblasts following bacterial challenge might attract immune cells into the odontoblast layer beneath the carious lesion [52]. Indeed, when dentin is being demineralised by caries, immature DCs accumulate at an early stage at the dentin-pulp interface in a strategic location to capture foreign antigens. A progressive and sequential accumulation of T cells (= T lymphocytes), macrophages, neutrophils, and B cells (= B lymphocytes) then occurs in the pulp, concomitantly with the deepening of the dentin lesion, the increase of the bacterial insult, and the development of the pulp inflammatory process [6, 53]. Thus, it is likely that odontoblasts are able to attract some, if not all, of these immune cell populations at the pulp-dentin interface to neutralize bacterial by-products that reach the pulpal end of the dentin tubules. By using culture supernatants of odontoblast-like cells stimulated with TLR2 agonists, we demonstrated that odontoblasts produced chemokines able to recruit immature DCs [12, 48]. CCL2, strongly expressed in odontoblasts beneath dentin carious lesions, may be involved in this process since it is a key element in the recruitment of circulating blood dendritic cells. Odontoblast-derived CXCL1, CXCL2, and CXCL8, which are known to attract neutrophils, and CXCL10, known to attract T cells, could be involved in the accumulation of other populations of immune cells at the dentin-pulp interface. However, to our knowledge, no direct evidence for a role of odontoblast-derived chemokines in these processes has been reported so far.

IL-6 is a pleiotropic cytokine produced by a variety of immune and nonimmune cells that regulates many aspects of the local immune response [54]. It is strongly upregulated in bacteria-challenged inflamed pulps* in vivo* and in odontoblasts* in vitro* upon TLR2 engagement [49, 55]. IL-6 is notably critical to the differentiation and regulation of T helper (Th)2, Th17, and T regulatory (Treg) phenotypes, and it promotes the secretion of acute-phase proteins including lipopolysaccharide-binding protein [19]. All these functions might be undertaken in inflamed pulps by IL-6. Since it also increases vascular permeability, IL-6 might also be involved in the formation of oedema induced by the progressive intradentinal penetration of Gram-positive oral bacteria [49].

IL-10 is an immunosuppressive cytokine produced by many immune and nonimmune cells which modulate immune responses to microbial antigens in order to prevent excessive or unnecessary inflammation. It acts in particular by decreasing the production of the proinflammatory cytokines IL-6 and CXCL8, thereby suppressing inflammation-associated immune responses and limiting damage to the host [56]. It also inhibits Th1 and Th2 immune responses but promotes the differentiation of regulatory T cells which control excessive immune responses in part by producing IL-10, which provides a positive regulatory loop for IL-10 induction [57, 58]. We found that IL-10 is upregulated in bacteria-challenged inflamed pulps* in vivo* [49] where it might help limit the spread of pulp inflammation which is initially restricted to the dentin-pulp interface beneath early dentin caries lesions [59]. IL-10 was upregulated in odontoblast-like cells* in vitro* upon TLR2 engagement, suggesting that odontoblasts are capable not only of initiating the pulp immune and inflammatory response to dentin-invading bacteria, but also of limiting its intensity [49].

Recently, we have studied the role of lipopolysaccharide-binding protein (LBP), an acute-phase protein known to attenuate proinflammatory cytokine production by activated macrophages. LBP has been shown to prevent the binding to host cells of several bacterial cell wall components including lipopolysaccharides, lipoteichoic acids, lipopeptides, and peptidoglycan [60]. It was also found to transfer lipopolysaccharides to high-density lipoproteins in the plasma for neutralization [61]. We recently detected LBP synthesis and accumulation in bacteria-challenged inflamed pulp, whereas this protein was not found in healthy pulp.* In vitro*, LBP was upregulated by Pam2CSK4 (a diacylated lipopeptide synthetic analog that binds specifically TLR2) in odontoblasts differentiated* in vitro*. It also decreased TLR2 activation and attenuated proinflammatory cytokine synthesis ([62], unpublished results). This molecule might be involved in the neutralization of bacterial components that gain access to the pulp, thus limiting activation of the pulp immune cells and the associated inflammatory response to dentin-invading bacteria [8].

**Figure 1:**
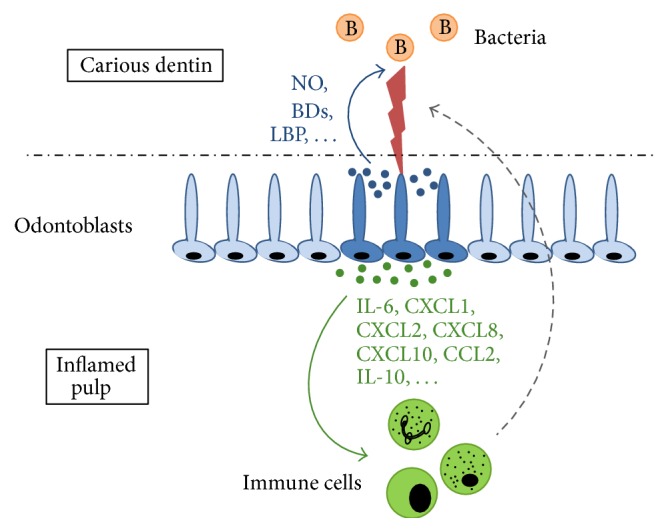
Two key aspects of the odontoblast defence against dentin-invading bacteria. Bacteria (B) present in the carious dentinal lesion release pathogenic components that activate (blue arrow) odontoblasts (dark blue) adjacent to the lesion, triggering the production of antibacterial molecules (blue dots). These molecules diffuse through dentin tubules in an attempt to destroy the invading microorganisms (NO, BDs) or considerably decrease their pathogenicity (LBP). In parallel, proinflammatory and immunomodulatory mediators (green dots), including IL-6, IL-10, CXCL1, CXCL2, CXCL8 (IL-8), CXCL10, and CCL2, are secreted by odontoblasts at the opposite cell pole and diffuse into the subodontoblast pulp area (green arrow) where they activate and mobilize various populations of immune cells (as described in the main text body) enabling the immunosurveillance of the tissue. Immune cells then migrate (dotted grey arrow) towards the pulp-dentin interface beneath the lesion to combat the bacteria and coordinate the immune defense response.

In summary, numerous studies performed over the last decade have shown that odontoblasts are able to detect oral microorganisms that invade mineralized dental tissues from the oral cavity. They mobilize themselves against this threat by building their own antibacterial arsenal (defensins, nitric oxide) and by sending molecular messengers (chemokines, cytokines) to the neighbouring pulp to alert immune cells able to mount responses to microorganisms (Figure 1). However, the majority of these studies have been performed* in vitro* and currently minimal information is available about the nature and role of antibacterial and immune effectors in caries-affected teeth* in vivo*. Additional experiments are therefore warranted to further characterize the molecular effectors and regulators of human dental pulp immunity and determine their therapeutic potential to promote the recovery of dental pulp homeostasis and health.

## 2. Response of Pulp Immune Cells to Tooth-Invading Pathogens

**Figure 2:**
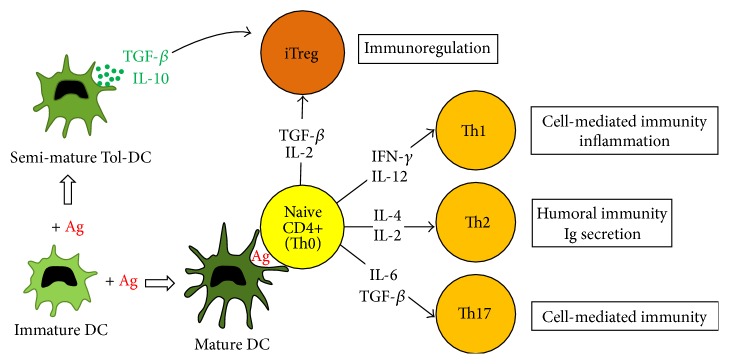
The putative role of dendritic cells (DCs) in the regulation of T helper (Th) and induced regulatory T (iTreg) cell differentiation. Upon encountering antigens (Ag), immature DCs usually become mature DCs which present antigens to naive CD4+ (Th0) cells. Upon antigen recognition, Th0 cells clonally expand and can differentiate into various subsets of effector cells (Th1, Th2, or Th17) or into iTreg cells depending on the cytokines present in their environment. Alternatively, immature DCs can mature only partially to become Tolerogenic-DCs (Tol-DCs) which can directly induce iTreg cell differentiation through TGF-*β* and IL-10 secretion. IL, interleukin; IFN, interferon; TGF, transforming growth factor; Ig, immunoglobulin.

As stated above, eliminating the decayed mineralized tissues containing microbial agents can result in decreased pulpal inflammation, promotion of tissue healing, and restoration of the normal biological functions of the pulp. Like peripheral organs and tissues such as skin, gastrointestinal tract, and lungs, healthy dental pulp contains sentinel leukocytes, which are able to biologically sample and respond to the local environment, including macrophages, DCs, and T cells [52, 53, 63, 64]. Fluorescence-activated cell sorting (FACS) analysis of enzymatically digested whole pulp tissue revealed that leukocytes represent ~1% of the total cell population in nonerupted human third molars [10]. Leukocytes in healthy tissue undertake immunosurveillance, that is, continuous sampling of their environment to sense microorganisms invading into the body. Their numbers significantly increase when pathogens are detected, due to the elevation of the inflammatory process. This inflammation is part of the normal protective immune response of the host to tissue infection and during this response, leukocytes from the circulatory system are triggered to adhere to endothelial cells lining blood vessels prior to them migrating out of the blood vessel to the site of infection. Neutrophils are initially recruited to the inflamed tissue to engulf and destroy invading microorganisms; subsequently this response is followed by monocytes which also differentiate into macrophages. In teeth, neutrophils and macrophages progressively infiltrate the pulp tissue as the carious disease progresses [4, 6, 9, 53, 65–67]. Macrophages are able to phagocytose bacteria and activate T cells triggering an adaptive immune response which occurs in association with DCs. In the pulp, DCs are initially present in an immature state and are attracted by odontoblast-derived chemokines to the site of infection, where they capture bacterial antigens diffusing through dentin tubules towards the pulp [6, 12, 48, 53]. Antigen uptake triggers the activation and progressive maturation of DCs, and they subsequently migrate to regional lymph nodes where they present antigens to, and activate, naive CD4+ T cells (also called Th0 cells). Activated DCs secrete a range of cytokines that influence both innate and adaptive immune responses, and they are considered key regulators of the tissue's defence against infection. Naive CD4+ T cells, when activated, can differentiate into effector CD4+ T helper cells or induced regulatory T (iTreg) cells [68]. Furthermore effector CD4+ T cells are classically assigned to Th1, Th2, or Th17 subsets and undertake specific functions in the immune response including regulation of cell-mediated immunity, inflammation, and protection against intracellular pathogens. Th1 cells are generated by IL-12 and interferon (IFN-) *γ* exposure and they secrete IFN-*γ*, IL-2, and TNF-*α*. Naive CD4+ T cells differentiate into Th2 cells following exposure to IL-4 and IL-2. Th2 cells produce IL-4, IL-5, IL-6, IL-10, IL-13, and IL-14; they regulate humoral (immunoglobulin-mediated) immunity and are involved in protection against extracellular pathogens. The Th17 lineage pathway provides a unique mechanism for protection against bacterial and fungal pathogens through the production and induction of inflammatory cytokines and the recruitment of neutrophils. Th17 cells are induced to differentiate from naïve CD4+ T cells mainly by transforming growth factor (TGF-) *β* and IL-6 [69] (Figure 2). We have previously provided precise quantification of T cells in healthy human dental pulp, enabling a better understanding of the initial capacity of the pulp to detect and combat pathogens. Our data demonstrated that cytotoxic CD8+ T cells represented ~21% total leukocytes, and CD4+ T cells were ~11%, with DCs ~4% of the leukocyte population. We observed that progressive and sequential accumulation of CD4+ and CD8+ T cells was observed in inflamed pulp which occurred in parallel with the deepening of the dentin lesion [4, 53, 67]. Elucidating the exact mechanisms that regulate Th1, Th2, or Th17 responses is essential to more comprehensively understand pulp pathogenesis; however to date no data are available regarding the subsets of T cells involved in these mechanisms. Thus far only one study has reported pulp regeneration in a mild irreversible pulpitis model after inhibition of IL-6 secretion by matrix metalloproteinase (MMP-) 3. The authors proposed that the control of IL-6 activities by MMP-3 could thus decrease the Th2 response and Th17 cell induction [70]. NK cells are also a well-known arm of the innate immune system. They are reported to exhibit features characteristic of the adaptive immune response and they have recently been identified in healthy rat molar pulps [71]. We have now found that NK cells represented ~2.5% of leukocytes in human healthy pulp [10]. In addition, a subset of T cells known as natural killer T (NKT) cells has been detected in healthy rat pulp [71] and these cells are known to play a major role in the development of Th1 versus Th2 immune responses [72]. Finally, a relatively small number of B cells are present in healthy pulp tissue and their numbers significantly increase during pulpitis and caries progression [10, 73]. Immunohistochemical analysis of inflamed pulp demonstrated that B cell-derived IgG1, rather than IgG2, is the dominant subclass of immunoglobulin followed by IgA and IgE [4, 65]. During human dental root resorption, B cells form clusters in the pulp of deciduous teeth [74] and their role may be to modulate DC functions [75].

In order to avoid irreversible damage to the pulp tissue, the complex immune responses must be controlled to enable pathogen destruction without causing damage to the host. Regulatory cells play a major role in this process [76]. In particular, subpopulations of immature DCs, called Tol-DCs, are resistant to maturation and are implicated in the regulation of the immune response [77]. They induce central and peripheral tolerance through different mechanisms including T cell depletion or anergy, induced Treg cell differentiation from naive CD4+ T cells, and production of a variety of immunomodulatory mediators such as PD-L1, PD-L2, heme oxygenase-1 (HO-1), HLA-G, galectin-1, DC-SIGN, IL-10, TGF-*β*, indoleamine 2,3-dioxygenase, IL-27, and NO [78, 79]. Naive CD4+ T cells differentiate into induced Treg cells (iTregs) following exposure to TGF-*β* and IL-2. They express CD4, CD25, and FoxP3 and secrete TGF-*β* and IL-35 that inhibit the effector T cell response. Among the iTreg population, Tr1 cells secrete a large quantity of IL-10 and TGF-*β* which suppress Th responses [80]. Relatively large numbers of iTregs have been detected in intensely inflamed human pulps [81]. FACS analysis, using healthy human molars, resulted in the detection of iTregs identified by the phenotype CD45+CD3+CD4+CD127low CD25+ and Foxp3+. There is also now evidence for the presence of a specific subset of DCs expressing HO-1 in healthy human pulp [10]. DCs expressing HO-1 have immunoregulatory properties, as this enzyme protects cells against inflammatory and oxidative stress [82]. Furthermore, myeloid derived suppressor cells (MDSCs) have been identified in healthy pulp and they constitute a heterogeneous population of cells with a remarkable ability to regulate immune responses [83–85]. Notably MDSCs expanded by exposure to bacterial components, such as lipopolysaccharide (LPS), regulate alloreactive T cells via HO-1 and IL-10 secretion [86]. Together, these results indicate that healthy dental pulp is equipped for limiting or fine-tuning innate and adaptive responses even in the absence of pathogens.

In summary, healthy dental pulp contains resident immune cells and is thus initially well equipped to detect and mount effective immune responses against invading pathogens. Recruitment of circulating immune cells into the pulp tissue during the inflammatory process reinforces its defence potential. In particular, it has recently been reported that the range of resident leukocytes is much wider in healthy pulp than previously understood and includes several populations of cells with immunoregulatory properties. These data indicate that the immune and inflammatory dental pulp response to pathogens is extremely complex. Additional studies are therefore warranted to understand how such a response can be controlled to promote tissue healing after pathogen removal by the dental practitioner.

## 3. Inflammation-Regeneration Interplay in the Dentin-Pulp Complex

Clearly, defence and reparative responses within the tooth are inextricably linked. During carious disease, which damages the tooth structure, the host aims to both fight the infection, via its immune-inflammatory response, and “wall off” and restore the tooth structure, via its dentinogenic responses.

Notably, the regenerative mechanisms within the dental tissues are underpinned and informed by developmental processes. Following a series of molecular and cellular signalling events which occur between the developmental epithelium and mesenchymal tissue, odontoblasts differentiate from progenitor cells bordering the dental papilla. In brief, they take on a polarised columnar form and secrete predentin and further signalling leads to cells of the inner enamel epithelium, which are in contact with the predentin, differentiating into polarised columnar ameloblasts, which subsequently synthesise the enamel. The predentin is converted to dentin and further cycles of predentin secretion and mineralisation result in the odontoblasts receding from the dentinoenamel junction towards the pulp core. As the dentin structure of the tooth develops, the odontoblasts leave their cellular processes extended within the dentinal tubules. A multitude of genes have been identified as being active during tooth development and morphogenesis, which indicates the complexity of the process [87]. Indeed, many of the growth factors involved in signaling the dentinogenic process subsequently become fossilised within the dentin as they are secreted by the odontoblast during development. Notably, their later release from the dentin during disease is understood to regulate both regenerative and defensive responses within the tooth and is discussed in more detail below.

Whilst primary dentinogenesis occurs at a rate of ~4 *μ*m/day of dentin deposition during tooth development, secondary dentinogenesis decreases to a rate of ~0.4 *μ*m/day following root formation and continues to occur throughout the life of the tooth. Tertiary dentinogenesis however describes the process of hard tissue repair and regeneration in the dentin-pulp complex, which is the tooth's natural wound healing response. With milder dental injury, such as early stage dental caries, primary odontoblasts become reinvigorated to secrete a reactionary dentin which is tubular and continuous with the primary and secondary dentin structures. However, in response to injury of a greater intensity, such as a rapidly progressing carious lesion, the primary odontoblasts die beneath the lesion [88, 89]. While it is not entirely clear what causes this odontoblast cell death, it is hypothesized that bacterial toxins, components released from the demineralised dentin or even local generation of high levels of proinflammatory mediators, signal this event. Subsequently, however, if conditions become conducive (e.g., if the carious infection is controlled or arrested), stem/progenitor cells within the pulp are signalled to home to the site of injury and to differentiate into odontoblast-like cells. These cells deposit a tertiary reparative dentin matrix, reportedly at a similar rate to that of primary dentinogenesis, and this clinically results in dentin bridge formation. The new hard tissue deposited walls off the dental injury and the infecting bacteria, protecting the underlying soft tissues, and partially restores tooth structure [90]. Clearly the relative complexity of these two tertiary dentinogenic processes differs, with reactionary dentinogenesis being comparatively simple and requiring only upregulation of existing odontoblast activity, whereas reparative dentinogenesis is more complex and involves recruitment, differentiation, and upregulation of dentin synthetic and secretory activity. Notably, it is understood that tertiary dentin deposition rates somewhat recapitulate those in development with dentin. Tertiary dentinogenic events are also understood to be signalled by bioactive molecules, similar to those present during tooth development. Some of these molecules may arise from the dentin when it is demineralised by bacterial acids as a variety of growth factors and other signalling molecules are sequestrated within the dentin during its deposition and formation [90–92]. The breakdown and release of signalling molecules from the dentin provide a means by which the tooth can detect tissue damage and subsequently rapidly respond. Indeed, an array of molecules are bound within dentin and are known to be released from their inactive state by carious bacterial acids, as well as restorative materials, such as calcium hydroxide, which are known to stimulate dentin bridge formation following clinical application. Furthermore a variety of molecules which in general are regarded as inflammatory mediators are also implicated in signalling repair responses. Clearly, it is likely that a fine balance exists between their levels and temporal and contextual profiles, which subsequently regulates the effects of these molecules on dental cells and tissues. These signalling aspects are further discussed below in more detail.

The carious infection, if unchecked, will progress through the dental hard tissues and into the soft pulpal core. In general, markers of the inflammation also subsequently increase including levels of cytokines and the immune cell infiltrate [64, 73, 93]. Indeed, the increased levels of cytokines have a range of regulatory functions including lymphocyte recruitment, extravasation, activation, differentiation, and antibody production. The roles of the cytokines, IL-1*α*, IL1-*β*, and TNF-*α*, are particularly well characterized in orchestrating the immune response in the pulp in response to carious and deeper associated periapical infections [93–100]. Initially, as has been discussed, resident pulp cells, including odontoblasts, will increase their expression of these molecules; however, a range of immune cells recruited to the lesion in response to infection will further add to the molecular milieu. Furthermore, components of dentin released by carious bacterial acids during the demineralization process have also been demonstrated to contribute to the levels of inflammatory mediators [101]. Notably, many other cytokines including IL-4, IL-6, IL-8, and IL-10 have been shown to be increased in pulp tissue, which is affected by carious disease [102–104]. It is a range of these potent cytokine signaling molecules which generates the chemotactic gradients leading to recruitment and activation of the immune cells described above and can subsequently lead to the chronic cycle of inflammation present within the tooth [105, 106].

Notably, the cytokine IL-8 is constitutively expressed by odontoblasts, likely in anticipation of disease events, and its levels can be significantly upregulated both by bacterial components (e.g., LPS via TLR signaling mechanisms) and by IL-1*β* and TNF-*α* in a range of cell types [107]. IL-8 is particularly important in the recruitment and activation of neutrophils, which are generally one of the first immune cell types present at the site of infectious disease (as described in detail above). Interestingly, we have reported elevated levels at both the transcript and protein levels for a range of proinflammatory mediators, including S100 proteins, in carious diseased pulpal tissue compared with healthy pulpal tissue [66, 93].

While local release and accumulation of proinflammatory mediators occur in response to the progressing carious infection, data now indicate that bacterial acid-driven dentin demineralization likely adds to the complex cocktail of signaling molecules present within the diseased dental tissue [66]. As we are aware that odontoblasts basally express certain cytokines [107], it is therefore perhaps of little surprise that these bioactive molecules become sequestrated within the dentin for later release when it is demineralised during the disease process. Indeed, the components of the dentin matrix are clearly multifunctional and can stimulate multiple processes such as promoting mineralization and stimulating cell migration and activation [92, 100, 101, 108].

The extravasation and antimicrobial activity of immune cells within the pulp result in the release of molecules that, while aimed at combatting the bacterial infection, can however also cause significant collateral host tissue damage. Degradative enzymes, such as MMPs necessary for the immune cell migration through the soft tissue matrix, cause degradative damage and the increased levels of reactive oxygen species (ROS) utilized by immune cells for antimicrobial action also damage host cells and tissues. These events can contribute to the chronic cycle of inflammation as these molecules are also known to have direct proinflammatory actions. Indeed, ROS, including superoxide anions, hydrogen peroxide, and hydroxyl radicals, can stimulate cytokine release by activating the key proinflammatory intracellular signaling pathways regulated by the p38 MAPK and NF-*κ*B proteins in several immune and tissue structural cell types [13, 109, 110]. Notably, these pathways have become exceedingly well characterized in the proinflammatory process and are central to extracellular signal transduction in response to cellular stresses, such as infection and cytokine stimulation [111, 112]. It should however be noted that while the activation of these signaling pathways is generally regarded as being involved in the amplification of the immune and inflammatory responses, they also appear to associate with repair and regeneration signaling. Indeed, while generally it is regarded that tissue repair does not occur until infection is under control and the inflammation is modulated, the magnitude and temporospatial nature of events may be key to fine-tuning this complex response. The link between inflammation and regeneration via these intracellular signaling interactions will be further discussed below.

Notably, the dentin-pulp complex has significant regenerative potential following injury due to its tertiary dentinogenic responses. Due to the differences in complexity of the cellular processes involved in reactionary or reparative dentinogenesis, the local inflammatory response will likely have differing effects at the different stages within it [66]. It is notable that tissue reparative events will likely only occur when the infection and inflammation are under control and this may result from the immune response resolving the infection, or following clinical intervention to remove the disease. This balance between defence and repair in the tissue is clearly important. Indeed, it would not appear practical for body resource to be utilized to rebuild tissue, which remains under attack from infection and hence may continue to break down. Furthermore, from a clinical standpoint, if the tissue is rebuilt while the infection is still present, this may prove futile and likely result in the need for retreatment.

In support of this premise, several lines of evidence indicate that chronic pulpal inflammation impedes reparative processes and the accepted paradigm is that regeneration only follows after appropriate resolution of inflammation, which likely occurs after disinfection [113–115]. Indeed, we know that while the immune-inflammatory responses aim to be protective, tissue damage occurs collaterally due to the release of degradative molecules and enzymes, as described above, and hence any reparative mechanisms ongoing may not be apparent. Potentially, the most significant evidence that resolution of infection and inflammation are necessary to enable regeneration is derived from classical animal studies, which demonstrated that repair was apparent only in artificial cavities made in germ-free animals compared with those where the cavities were infected and subsequent inflammation occurred [116]. Further evidence regarding the effects of inflammation on regeneration comes from* in vitro* studies that demonstrate the biphasic responses of pulp cells to proinflammatory signaling molecules. Notably, while relatively low levels of cytokines and growth factors can be stimulatory to cells, high levels of these molecules, such as TNF-*α* and TGF-*β*, present during infection and inflammation can cause cell death [97, 108, 117, 118]. More direct evidence also comes from studies that demonstrate stem cell differentiation processes are clearly impeded by proinflammatory signaling [119, 120].

Recent work has, however, indicated that inflammatory signals can stimulate repair processes (reviewed in [121]). Indeed, signal transduction via both the key proinflammatory MAPK and NF-*κ*B pathways (as described above) is also implicated in several reparative response processes. Data from several sources have demonstrated that these intracellular cascades can be activated in dental cells by several inflammation-related molecules, including bacterial components, ROS, and cytokines, which subsequently drive* in vitro* mineralization and differentiation responses. Arguably, it may be that acute or low levels of these inflammatory signals are necessary to signal these regenerative responses [109, 122–128]. Interestingly, it is also known that dying cells release and promote local secretion of low levels of proinflammatory mediators as damage-related signals [129]. Potentially, this sterile inflammation may occur during pulpal fibroblast senescence in the aging pulp and, subsequently, this process may generate nucleation points which drive pulp stone formation [130]. Combined, these data indicate that a delicate balance exists between the signaling or inhibition of repair and regeneration by proinflammatory mediators. Subsequently, we hypothesize that relative low level or acute inflammation may stimulate tissue regeneration, whilst higher chronic levels may impede the reparative processes and favor intense immune cell recruitment and activation.

Intriguing evidence linking the two processes of repair and regeneration can also be derived from data which demonstrates the sharing of receptors between immune and repair-related cells. Indeed, the C-X-C chemokine receptor 4 (CXCR4) is known to be expressed on both of these two different cell types [131, 132]. Furthermore, both the receptor and its ligand, stromal cell-derived factor-1 (SDF-1)/CXCL12, have been detected within the dentin-pulp complex and are reportedly upregulated during dental disease [133, 134]. Potentially, the sharing of this chemotactic receptor by these cell types appears somewhat logical as tissues which are damaged or infected, as is the case with the tooth during caries infection, need to recruit both immune and stem cells to injury sites to facilitate defence and repair [135]. The regulation as to which of these two processes predominates may, however, be locally controlled as studies have shown that cytokine levels modulate the stem cell surface expression of CXCR4. It is therefore conceivable that relatively high levels of proinflammatory molecules may abrogate CXCR4-mediated stem cell response at sites where inflammation is overriding [131].

Further support for the role of inflammation events preceding repair is potentially provided clinically following the application of the chemically related pulp capping agents of calcium hydroxide and Mineral Trioxide Aggregate (MTA). These restorative agents are known to enable the formation of tertiary dentin, in the form of a dentin bridge, beneath the site of application. Notably, however, chronologically prior to visible signs of hard tissue healing process, dental tissue inflammation is routinely observed histologically [136]. While calcium hydroxide has been applied clinically for over 60 years [137–140], its mechanism of action in the induction of reparative dentinogenesis remains controversial, although its beneficial effects have been attributed to the local release of hydroxyl ions [139], which raise pH and lead to cellular necrosis [141, 142]. Hence, it is the nonspecific chemical tissue irritation effect of these restoratives which has been cited as their principal mechanism of action for promoting dentin-pulp complex tissue regeneration. More recent studies have also indicated that these regenerative effects are perhaps more related to their ability to sterilize the site of infection whilst releasing bioactive signaling components from the dentin [143, 144]. It could therefore be hypothesized that a combination of events may occur to facilitate dentin-pulp complex repair* in vivo* following their placement. Indeed, the local cellular necrosis may stimulate sterile inflammation [145–148], which is able to resolve due to the elimination of bacteria by the combination of the material and clinical procedure. This relatively mild and acute immune response combined with the leaching of growth factors and signaling molecules from the dentin may subsequently generate a conducive environment for reparative dentinogenesis [149–152]. Furthermore, it has been observed that MTA can increase cytokine release, including IL-1*α*, IL-1*β*, IL-2, IL-6, and IL-8, from mineralizing cells and this mild and acute material-induced inflammatory response may also contribute to clinical repair [153–155].

**Figure 3:**
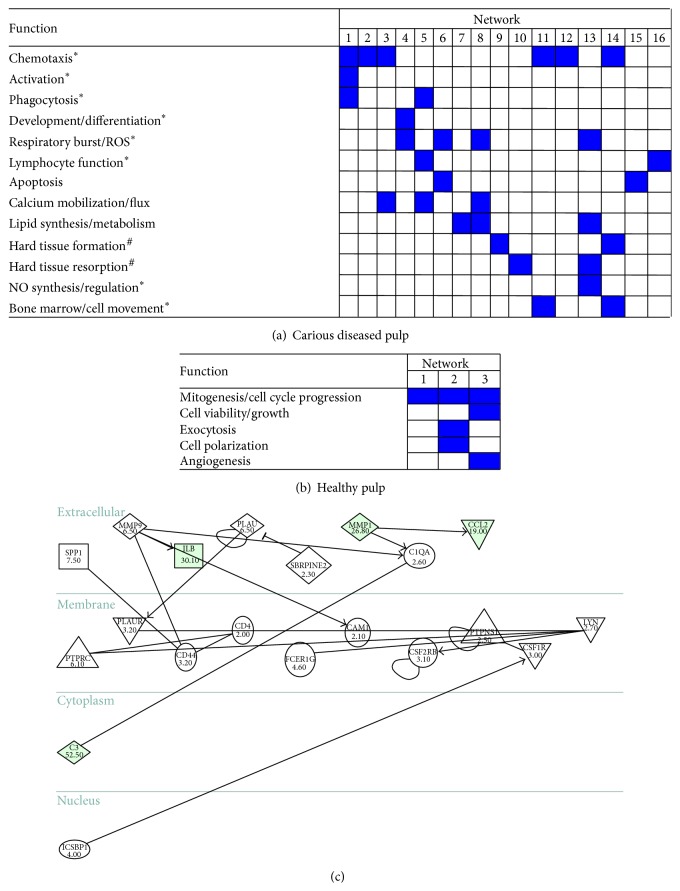
Tables ((a) and (b)) showing the key functions associated with the 16 and 3 molecular networks identified as being significantly activated (≥ 6 focus genes) in carious and healthy pulpal tissue, respectively. Shading of boxes indicates the networks which associated with the function and hence supported its inclusion as being active. Analysis was performed using the Ingenuity Pathways Analysis (IPA) software (http://www.ingenuity.com/products/ipa) on the high-throughput datasets reported in McLachlan et al. [11]. Sixteen and three functional categories were identified as being activated in carious diseased and healthy pulpal tissues, respectively. Carious diseased pulp tissue clearly demonstrated increased molecular network and functional activity compared with healthy pulpal tissue. Asterisks (*∗*) in (a) indicate functions which are associated with immune system cells (as identified by IPA); notably some evidence of hard tissue repair function was also evident (#). Ontological functions identified in (b) likely associate with pulp tissue homeostatic processes. Image (c) shows an example network (network 1 from the carious pulp tissue dataset) which also shows the subcellular localisation of the molecules that were identified as differentially expressed. The activation of this network via intracellular signalling cascades results in the elaboration of key inflammatory-associated chemokines, such as CXCL8 (IL-8) and CCL2, and the matrix metalloproteinases (MMPs) 1 and 9.

To better characterise the molecular response of the pulp tissue during caries, we have undertaken high-throughput transcriptional profiling using disease and healthy pulp tissue. Data indicated that the predominant tissue processes, pathways, and molecular interactive networks detected were proinflammatory in nature, while there was minimal evidence of repair-associated molecular events [11] (Figure 3). Indeed, increased expression of many well-characterised proinflammatory mediators was detected while further data-mining enabled us to identify expression changes in several molecules previously not associated with dental tissue disease. We subsequently speculated that underlying molecular repair-related responses may be occurring and, therefore, further bioinformatically interrogated our datasets and identified the candidate repair-related molecule, adrenomedullin (ADM). This pleiotropic cytokine was upregulated during dental disease and is reported to have antibacterial and immunomodulatory properties, as well as being a known molecular mediator of angiogenic and mineralized tissue reparative processes. Others have also shown that it is able to modulate inflammation at the molecular level [156–159]. Our subsequent studies went on to demonstrate that ADM may exert similar effects within the dental tissues and is archived within the dentin during primary dentinogenesis [160]. These data indicate that this molecule may be a viable target for use in future biological therapies for both hard and soft tissue repair of the dentin-pulp complex.

While it is aimed at identifying molecular modulators of dental tissue inflammation, which may have efficacy in enabling hard tissue repair, it is also interesting to speculate that direct delivery of mesenchymal stem cells (MSCs) or their secretomes may provide a novel approach to control inflammation. Indeed, adult/postnatal MSCs, including dental pulp stem cells, isolated from a range of tissues have demonstrable immune-modulatory capability either via their cell-cell contact or via their secreted components which can inhibit proliferation, cytokine/antibody secretion, immune cell maturation, and antigen presentation by T cells, B cells, NK cells, and DCs [161–163]. Direct cell-to-cell contact between stem and immune cells is known to elicit secretion of soluble factors such as TGF-*β*1 and indoleamine-2,3-dioxygenase-1 which subsequently can dampen the immune response. While MSCs may provide a cell therapy approach to aid repair of inflamed dental tissue if delivered appropriately, better characterization of their secreted active components may enable identification of novel molecules for targeted dental tissue repair.

Data now indicate that, during a progressive carious infection, initially it is the odontoblasts which detect the invading bacteria and, subsequently, cells within the pulp core such as resident immune cells, fibroblasts, stem cells, and endothelial cells become involved in the molecular response. Further autocrine and paracrine signalling amplifies the reaction and leads to an increased immune cell infiltration. The elaboration of a plethora of cytokines and chemokines will have resultant consequences for the tissue and its innate repair mechanisms and this milieu is further added to by the signalling molecules released from the dentin matrix itself by the action of bacterial acids [48]. This local cocktail of bioactive molecules will continue to chronically recruit and activate immune cells, which combat the invading bacteria. The relatively high levels of proinflammatory mediators present in the local environment will likely impair any healing events at the cellular and molecular levels. Currently, the application of dental clinical procedures and restorative materials aims to remove the infection, facilitate the resolution of the inflammatory response, and enable repair processes. Notably, attempts are now being made to apply knowledge of the cytokine networks invoked for diagnostic and prognostic purposes. It is envisaged that these data will enable identification of lesions refractory to endodontic treatment due to unresolved chronic inflammation [164].

While diagnostics are being developed based on the characterisation of the inflammatory response, modulators of inflammation have the potential to be used adjunctively to facilitate the healing response and aid restoration longevity. Recent work has demonstrated that dental resin restorative procedures can be supplemented with antioxidants, such as N-acetyl-cysteine (NAC). This supplementation reportedly provides protection to the pulpal cells from ROS generated following resin placement. Interestingly, NAC may also limit the activation of the key ROS activated NF-*κ*B proinflammatory pathway [165] and this modulation may also minimise the inflammatory response, subsequently creating a more conducive environment for tissue repair. More studies in this area may identify other antioxidants and pathways, which may facilitate dental tissue repair responses.

Other work has demonstrated the importance of the modulation of both ROS and reactive nitrogen species (RNS) to facilitate repair. Kim et al. [166] have recently demonstrated that the anti-inflammatory mechanism of exogenously applied PPAR*γ* in activated human dental pulp cells was likely due to the removal of both NO and ROS, which subsequently suppressed both the NF-*κ*B inflammatory and extracellular signal-regulated kinase (ERK) 1/2 signaling pathways. The anti-inflammatory effects of other naturally derived compounds, such as pachymic acid, derived from the mushroom* Formitopsis niagra,* have also been explored. Interestingly, this compound may not only have anti-inflammatory activity, but also appears to be able to promote odontoblast differentiation via activation of the HO-1 pathway. These data further indicate the important interrelationship between inflammation and repair and its potential application for dental disease treatment [167]. Recently, an exciting area relating to the therapeutic application of regulatory microRNAs (miRNAs) has been reported. These miRNA molecules have been shown to be differentially expressed between healthy and diseased dental pulps [168] and work is ongoing within the pharmaceutical industry to engineer these molecules for delivery to treat a range of inflammatory diseases. Potentially, miRNAs may therefore one day be applied in the treatment of dental disease as a means to tip the balance from a chronic inflammatory environment to one more conducive for tissue repair. It is now evident that more studies are required which target the interactions between the inflammatory and regenerative responses within the dentin-pulp complex as these may identify novel therapies for dental tissue repair.

## 4. Conclusion

We are now developing a better and more complete understanding of the molecular and cellular events which occur in the dentin-pulp complex during inflammation and repair following carious disease. While disinfection of the dental tissue is clearly imperative for the health of the tooth, the subsequent interaction between dental tissue defence and repair is complex and the fine-tuning of the regulation of these processes is important for ensuring which response predominates when vital pulp tissue can be clinically retained or regenerated. It is clear that sustained research activity in this area combined with clinical translational approaches may result in the development of new therapeutics which enable host defence and repair events. Advances in our understanding of the interactions between immune and regenerative responses may therefore influence clinical practice and benefit dental patients in the future.
